# Visual search performance in infants associates with later ASD diagnosis

**DOI:** 10.1016/j.dcn.2016.09.003

**Published:** 2016-09-30

**Authors:** C.H.M. Cheung, R. Bedford, M.H. Johnson, T. Charman, T. Gliga, the BASIS team

**Affiliations:** a Centre for Brain and Cognitive Development, Birkbeck, University of London, UK; b Biostatistics Department, Institute of Psychiatry, Psychology & Neuroscience, King’s College London, UK; c Department of Psychology, Institute of Psychiatry, Psychology & Neuroscience, King's College London, UK

**Keywords:** Visual search, Visual attention, ASD, ADHD, Infant, Familial risk

## Abstract

An enhanced ability to detect visual targets amongst distractors, known as visual search (VS), has often been documented in Autism Spectrum Disorders (ASD). Yet, it is unclear when this behaviour emerges in development and if it is specific to ASD. We followed up infants at high and low familial risk for ASD to investigate how early VS abilities links to later ASD diagnosis, the potential underlying mechanisms of this association and the specificity of superior VS to ASD. Clinical diagnosis of ASD as well as dimensional measures of ASD, attention-deficit/hyperactivity disorder (ADHD) and anxiety symptoms were ascertained at 3 years. At 9 and 15 months, but not at age 2 years, high-risk children who later met clinical criteria for ASD (HR-ASD) had better VS performance than those without later diagnosis and low-risk controls. Although HR-ASD children were also more attentive to the task at 9 months, this did not explain search performance. Superior VS specifically predicted 3 year-old ASD but not ADHD or anxiety symptoms. Our results demonstrate that atypical perception and core ASD symptoms of social interaction and communication are closely and selectively associated during early development, and suggest causal links between perceptual and social features of ASD.

## 1 Introduction

Enhanced perceptual abilities have repeatedly been described in individuals with autism spectrum disorder (ASD) (for a review, Mottron et al., 2006). For example, better performance has been reported in visual search paradigms, which measure the speed or detection accuracy of “odd-one-out” target elements presented amongst arrays of distractors (for a review see Kaldy et al., 2016). The mechanisms underlying the perceptual advantage in ASD are yet poorly understood. According to one hypothesis, the Weak Central Coherence theory, the superior ability to detect or discriminate visual features is a byproduct of the poor ability to attend to the higher-level, semantic information in visual scenes (Frith, 1989, Happé and Frith, 2006). This would explain, for example, why people with ASD can as easily find a geometric figure embedded in a meaningful or in a meaningless drawing, while control participants are slower in the former condition, when they prioritize the overall meaning (e.g. Jolliffe and Baron-Cohen, 1997). However, others have shown superior perceptual performance in tasks employing stimuli without semantic content, such as the visual search task. Across many variations of these paradigms, individuals with ASD are both quicker and more successful than controls at detecting abstract targets, as for example a letter X presented amongst Os (Joseph et al., 2009, Kaldy et al., 2011, Plaisted et al., 1998). These studies suggest that atypicalities might be present at the earliest stages of sensory or perceptual processing in ASD as is also suggested by recent evidence for better discrimination of line orientations (Dickinson et al., 2014), increased orienting to pixel-level saliency in participants with ASD than in neurotypicals (Wang et al., 2015), as well as by evidence for superior pitch discrimination and memory (Heaton et al., 2008, Stanutz et al., 2014)

These sensory or perceptual atypicalities pose a challenge for understanding its etiology of ASD. Whether and how these features relate to core social and communication difficulties remains a contentious issue (Happé and Frith, 2006). Several studies carried out in older children or adults with ASD failed to measure an association between superior perception and social cognition tasks or social skills (e.g. Cantio et al., 2016, Morgan et al., 2003, Pellicano et al., 2005), while others did find an association, for example between line orientation discrimination and autism quotient scores (Dickinson et al., 2014). More generally, dimensional measures of social and non-social symptoms are poorly correlated (Happé and Ronald, 2008), and there is evidence for reduced genetic overlap, indicating that perceptual and social atypicalities might be independent aspects of ASD (Ronald et al., 2005). However, evidence for the fractionation of the autism phenotype comes mainly from research carried out with older children and adults. An alternative view is that atypical perception and social skills are intrinsically related during early development but may diverge later due to adaptive changes specific to each domain (Gliga et al., 2015b). In support of this hypothesis, we showed that in younger siblings of children with ASD, improved search performance at 9 months, associates with ASD symptom severity at 15 months and 2 years of age (Gliga et al., 2015a). At 2 years of age, autism symptoms no longer related to concurrent search performance.

This initial work left several key questions unanswered. First, does superior performance in visual search during infancy discriminate those children that go on to receive a clinical diagnosis of ASD from the other high risk children and control participants? Second, what drives superior search performance; is it due to better discrimination abilities or better attention to the task? Increased arousal was shown to associate with better performance in visual search in toddlers with ASD (Blaser et al., 2014) and might be a common driver of both superior perception and social interaction atypicality. Finally, does superior perception specifically predict ASD symptoms as opposed to other aspects of early emerging psychopathology? Building theoretical models that link perceptual and social atypicalities in ASD will greatly benefit from evidence that these features are selectively associated.

We investigate these issues in a cohort of infants at familial risk for ASD (who have an older sibling with the disorder). About 20% of younger siblings develop ASD themselves (Ozonoff et al., 2015) and another 20% will manifest subthreshold symptoms or developmental delay (Messinger et al., 2015). Infant sibs research has yielded a variety of infancy markers of later clinical autism, indexing a broad spectrum of putative neural systems, such as attention control (Elsabbagh et al., 2013, Elsabbagh et al., 2009, Wass et al., 2015), face and gaze processing (Chawarska et al., 2013, Chawarska et al., 2010, Elsabbagh et al., 2015, Jones et al., 2014) or motor planning (Flanagan et al., 2012). A specific impairment of the “social brain” circuitry no longer seems the most parsimonious explanation of these findings (Johnson et al., 2015) and domain general mechanisms have been suggested as developmental pathways to ASD (Gliga et al., 2015b, Varcin and Nelson, 2016). Showing that superior perception is one of the earliest markers of ASD in high-risk populations would support this emerging view. The current paper builds on a previous publication (Gliga et al., 2015a), with an extended sample of participants (34 additional high-risk participants), followed-up at 3 years of age. We report on the association between visual search in infancy and ASD diagnosis at 3 years of age. In addition, we investigate the impact that target/distractor similarity and attention to the task, have on performance. Finally, associations to dimensional measures of ASD, attention- deficit/hyperactivity disorder (ADHD) and anxiety symptoms at age 3 years are also assessed.

## 2 Methods and materials

### 2.1 Participants

A cohort of 116 high-risk (HR) (64 male; 52 female) and 27 low-risk (LR) children (14 male; 13 female) participated in this longitudinal study. All HR children had at least one older sibling with a community clinical diagnosis of ASD (see Supplemental Online Material (SOM) for details). LR controls were full term infants (gestational ages 38–42 weeks) recruited from a volunteer database at the Birkbeck Centre for Brain and Cognitive Development. Families attended four visits at 9, 15, 27 and 36 months. Three HR children did not take part in the 36-month visit and they were excluded from the analysis. Two LR children were absent in the 36-month visit but were included in the analysis as they showed typical development at the previous three visits. The final sample included in this analysis consisted of 113 HR and 27 LR children (further exclusion criteria based on data availability/quality are presented in Section 2.4).

### 2.2 Clinical measures

A battery of clinical research measures was administered to all children at 36 months: the *Autism Diagnostic Observation Schedule – Second Edition* (*ADOS-2*; Lord et al., 2012), a standardised observational assessment, was used to assess current symptoms of ASD (116 children were administered Module 2 and 20 children Module 1, ADOS not completed with 5 HR and 2 LR children). Calibrated Severity Scores for Social Affect, and Restricted and Repetitive Behaviours (RRB) were computed (Gotham et al., 2009), which provide standardised autism severity measures that account for differences in module administered, age and verbal ability. The *Autism Diagnostic Interview – Revised* (*ADI-R*; (Le Couteur et al., 2003), a structured parent interview, was completed with parents of all children. Standard algorithm scores were computed for Reciprocal Social Interaction (Social), Communication, and Restricted, Repetitive and Stereotyped Behaviours and Interests (RRB). These assessments were conducted without blindness to risk-group status by or under the close supervision of clinical researchers (i.e., psychologists, speech therapists) with demonstrated research-level reliability. Total scores of the *Social Communication Questionnaire* (SCQ; Rutter et al., 2003) were used as additional parent-report measures of ASD symptoms. The parent-reported *Child Behavior Checklist* (CBCL/1.5–5; Achenbach and Rescorla, 2000) was used to measure clinical-levels of ADHD and anxiety problem, computed by T-scores for ADHD and anxiety problems from the DSM-oriented scales. This measure has been widely used to measure emerging psychopathologies in young children (Rietveld et al., 2003). We used the early learning composite score of the *Mullen Scales of Early Learning* (MSEL; Mullen, 1995) to obtain a standardised measure of mental abilities at every visit.

Experienced researchers (TC, GP, CC) reviewed information on ASD symptomatology (ADOS-2, ADI-R, SCQ), adaptive functioning (*Vineland Adaptive Behavior Scale-II*, (Sparrow et al., 2005), and development (MSEL) for each HR and LR child to ascertain ASD diagnostic outcome according to DSM-5 (American Psychological Association, 2013). Of the 113 HR participants included in this paper, 17 (15 boys, 2 girls) met criteria for ASD (hereafter, HR-ASD). A further 32 participants (20 boys, 12 girls) did not meet ASD criteria, but were not considered typically-developing, due either to a) scoring above ADI-R cut-off for ASD (Risi et al., 2006) and/or scoring above ADOS-2 cut-off for ASD (n = 18), or b) scoring less than 1.5 SD below the population mean on the Mullen Early Learning Composite (<77.5) or on the Mullen Expressive Language or Receptive Language subscales (<35) (n = 9), or meeting both of points a and b above (n = 5). These participants therefore comprised a HR subgroup, who did not meet clinical criteria for ASD but presented with other atypicalities (hereafter, HR-ATYP). The remaining 64 HR participants (28 boys, 36 girls) were typically-developing (hereafter, HR-TD). None of the 27 LR children (14 boys, 13 girls) met DSM-5 criteria for ASD and none had a community clinical ASD diagnosis. Descriptive characteristics and clinical measures for each group are presented on Table 1.

**Table 1:** Detailed characterisation of HR subgroups and LR controls at all visits for all participants that contributed visual search data.

	**High risk**			**Low risk**	p values
	ASD	Atypical	Typical		
**Visit 1**–9 months					
Age in days (SD) MSEL ELC N (% boys)	268.59 (24.99) 104.12 (16.93) 17 (88.24)	275.32 (23.05) 101.9 (14.12) 31 (61.29)	276.89 (26.04) 110.19 (15.03) 64 (43.75)	282.70 (25.56) 112.63 (13.32) 27 (51.85)	ns .07
**Visit 2**–15 months					
Age in days (SD) MSEL ELC N	465.38 (32.29) 83.38 (12.20) 13	468.38 (31.62) 91.69 (17.37) 29	468.17 (28.71) 98.24 (12.54)^a^ 64	471.35 (24.29) 104.19 (15.46)^a^ 26	ns <.01
**Visit 3**–2 years					
Age in days (SD) MSEL ELC N	811.35 (82.88) 81.25 (20.32) 17	799.59 (54.06) 93.75 (23.99)^a^ 27	802.21 (42.73) 104.02 (15.46)^a^ 58	768.19 (33.48)^a^ 114.54 (14.85)^a^ 26	.01 <.01
**Visit 4**–3 years					
Age in months (SD)	38.56 (1.71)	38.69 (1.87)	38.92 (1.45)	38.72 (1.62)	ns
MSEL ELC	84.81(28.20)	87.44(25.59)	114.57 (15.89)^a^	119.48 (15.26)^a^	<.01
ADI-Social	12.13 (5.76)	3.06 (3.16)^a^	1.46 (2.00)^a^	.96 (1.48)^a^	<.01
ADI-Communication	11.50 (4.69)	4.44 (4.26)^a^	1.70 (2.19)^a^	.48 (1.05)^a^	<.01
ADI-RRB	5.63 (2.55)	1.25 (2.16)^a^	0.46 (0.91)^a^	.08 (0.28)^a^	<.01
ADOS-Social	4.13 (3.12)	4.48 (2.41)	1.58 (0.75)^a^	2.56 (1.96)^a^	<.01
ADOS-RRB	6.25 (1.61)	5.29 (2.68)	3.37 (2.33)^a^	3.48 (2.31)^a^	<.01
SCQ	16.93 (6.82)	6.67 (6.78)^a^	3.75 (4.12)^a^	2.67 (2.33)^a^	<.01
CBC ADHD	63.60 (9.37)	57.03 (8.37)^a^	53.09 (5.58)^a^	51.04 (2.90)^a^	<.01
CBC Anxiety	60.47 (11.72)	55.93 (8.56)	52.86 (6.64)^a^	51.08 (2.19)^a^	<.01

^a^indicates significant differences with the HR-ASD group.

MSEL ELC – Mullen Scales for Early Learning Early Learning Composite; ADI – Autism Diagnostic Interview; ADOS – Autism Diagnostic Observation Schedule; SCQ – Social Communication Questionnaire; CBC – Child Behavior Checklist.

### 2.3 Stimuli and procedure

**Fig. 1:**
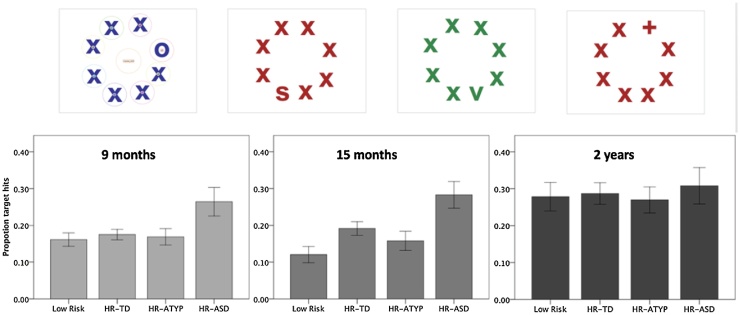
Proportion of correct first looks, at 9 months, 15 months and 2 years. Bars represent 1 SE.

We created arrays of eight letters, situated on an imaginary circle, and on a white background (Fig. 1). In each array, seven of the stimuli were letters “x” (the distractors), the 8th stimulus was either “+”, “v”, “s” or “o” (the targets). For the 9- and 15- month visit, 8 different arrays were created for each target type varying in the position of the target, generating 32 different stimuli in total. To increase variability, letters in an array were either black, blue, red or green (25% of arrays for each colour). Due to time constraints, only 50% of the stimuli were presented at the 27-month visit, generating 16 trials in total (4 trials for each target type) (Gliga et al., 2015a). At all visits this task was the first to be administered after parents and baby were welcomed to the lab, and was followed by a battery of eye-tracking tasks.

Infants were seated on mother’s lap, at approximately 60 centimetres from a Tobii T120 screen. A five-point calibration routine was run. The experiment was started only after at least 4 points were marked as being properly calibrated for each eye. The infant’s behaviour was monitored by a video camera placed above the Tobii monitor. Stimuli were presented with Tobii Studio software. Each of the stimuli was presented once, in a random order, for 1.5 s. Before each stimulus the child’s attention was directed to the centre of the screen using a 1 s long audio-video animation (attention getters).

### 2.4 Statistical analyses

At 9 months, one HR-ATYP participant was excluded due to eye-tracking equipment failure. At 15 months, 8 infants did not contribute data, one because they did not attend the lab visit (HR-ATYP), two HR-ASD participants were excluded due to eye-tracking equipment failure and for five infants the task was skipped due to fussiness (LR n = 1; HR-ATYP n = 2, HR-ASD n = 2). At 24 months, 5 toddlers (HR-ATYP) did not take part in the visit and for 6 others (HR-TD) the task was skipped due to fussiness.

We first explored whether outcome groups differed in general attention to task, to ensure that this does not account for any group differences in visual search performance itself. Subsequently, our primary analyses used repeated measures ANOVA to test outcome group differences in first-look hits, calculated as the proportion of trials in which infants made a first saccade towards one of the targets, after fixating at the centre of the screen. Between the 9- and 15-month visits, 54 (47%) of the high-risk families took part in a randomised controlled trial (RCT) of parent-mediated intervention (Green et al., 2015), with an additional six families enrolled in a similar non-RCT intervention (Green et al., 2013). Preliminary analysis accounted for the fact that some of the participants were taking part in these intervention programmes (see SOM for more detail). As there were no significant effects of either recruitment (being enrolled in the intervention, irrespective of whether the children were in treatment or control group) or the intervention itself (i.e. being in the treated arm of the RCT intervention or in a non-RCT intervention), we removed these factors from further analysis.

A first set of ANOVAs was run without additional covariates and followed up by post-hoc *t*-tests comparing performance of the HR-ASD groups against all other groups. Covariates (e.g. chronological age at test and sex) were entered in a second round of analyses. When at a particular age a significant effect of group was found, we carried out additional analyses to further understand the mechanisms driving these effects (see Section 3.3 and the SOM). Descriptive statistics for number of valid trials and first-look hits are presented in Table 2.

**Table 2:** Mean and standard deviation of the number of valid trials and proportion of first-look target hits for each outcome group.

	HR-ASD	HR-ATYP	HR-TD	Low Risk
**Visit 1**–9 months				
Valid trials (SD) N Target hits (SD) N	16.24 (2.63) 17 .26 (.16) 17	13.58 (4.05) 31 .17 (.12) 29	12.91 (4.57) 64 .17 (.11) 58	12.22 (4.98) 27 .14 (.09) 27
**Visit 2**–15 months				
Valid trials (SD) N Target hits (SD) N	13.00 (6.64) 14 .28 (.12) 11	13.97 (6.10) 29 .16 (.13) 26	13.00 (5.21) 64 .19 (.14) 57	12.31 (5.18) 26 .11 (.10) 25
**Visit 3**–2 years				
Valid trials (SD) N Target hits (SD) N	10.88 (3.18) 17 .31 (.18) 15	10.74 (3.58) 27 .28 (.15) 20	9.71 (4.04) 58 .29 (.19) 44	9.76 (3.17) 26 .26 (.18) 24

## 3 Results

### 3.1 Attention to the task

A trial was considered valid if the participant made a first saccade to the centre of the display, within 100 ms from the beginning of the trial. Number of valid trials was entered in separate univariate ANOVAs for each age group.

At 9 months, there was a main effect of the outcome group (F(3,135) = 3.34, p = 0.021 η^2^ = 0.07). Post-hoc pairwise *t*-tests (Dunnett t) comparing all groups against HR-ASD yielded significant differences with LR (p = 0.005), HR-TD (p = 0.015) and no difference with HR-ATYP (p = 0.10). When MSEL, age and sex were entered as covariates, the effect of outcome group remained significant (F(3,135) = 2.70, p = 0.049, η^2^ = 0.058), with no significant effects of the covariates (ps > 0.56).

The group difference in the number of valid trials found at 9 months merited additional analysis to clarify their origin. These analyses are detailed in the SOM. In brief, at 9 months groups differed in the amount of looking time to the stimuli (F(3, 135) = 3.356; p = 0.021; η^2^ = 0.069), with the HR-ASD group showing longer looking time than the LR group (p = 0.008). Bivariate correlations indicated that the number of valid trials were significantly associated with the total looking time to the stimuli (r = 0.81, p < 0.01). This suggests that both these variables reflect differences in sustained visual attention, with the HR-ASD group showing better attention to the task.

At 15 months, outcome groups did not significantly differ in the number of valid trials contributed to the analysis (F(3,127) < 1). There were also no main effects of age, sex or MSEL, in the follow-up analysis (Fs(1,123) < 1).

At 2 years, the same pattern was observed, with no significant effect of outcome group (F(3,124) < 1) nor of any of the covariates (MSEL, age or sex (Fs(1,116) < 1)).

### 3.2 First look target hits

To analyse how groups differed in the proportion of first look hits, we ran repeated measures ANOVA with target types (o, s, +, v) as the within-subject factor, and outcome group between subjects. This effectively means that a minimum of 4 trials was required for a participant to contribute to the analysis. At 9 months, this analysis yielded a main effect of trial type (F(3,369) = 15.944, p <0.001, η^2^ = 0.115; Fig. S1). All groups performed better in the ‘o’ and ‘s’ targets trials than the ‘v’ and ‘ + ’ targets trials (see SOM). There was also a main effect of group (F(3,123) = 3.352, p = 0.021, η^2^ = 0.076; Fig. 1) but no significant interaction between trial type x group (F(9,369) = 1.274, p > 0.1). Post-hoc *t*-tests indicated that HR-ASD had a significantly higher proportion of first looks to the target than LR (p = 0.014), HR-TD (p = 0.015) and HR-ATYP group (p = 0.019; Fig. 1). First look hits did not correlate with the quantity of valid trials (Pearson’s r = 0.123, p < 0.1; Fig. S2). However, because a group difference in the quantity of valid trials was observed at this age, this measure was entered together with MSEL, sex and age as covariates, in the follow-up ANOVA. The main effect of group remained significant (F(3,119) = 2.80, p = 0.043, η^2^ = 0.066) and none of the covariates had a significant impact on target hits (Fs(1,119) < 1).

At 15 months, a main effect of outcome group was again observed (F(3,113) = 4.15, p = 0.008, η^2^ = 0.099; Fig. 1), alongside a main effect of trial type (F(3,339) = 15.324, p < 0.001; Fig. S1) and no significant interaction between trial type x group (F(9, 339) < 1). At this age, also, HR-ASD demonstrated superior performance when compared to HR-ATYP (p = 0.030) and LR (p = 0.002) and marginally, when compared to HR-TD (p = 0.077; Fig. 1). When age, sex and MSEL were added as covariates, the effect of group became marginal (F(3,109) = 2.212, p = 0.091, η^2^ = 0.057). MSEL had a significant effect on performance (F(1,109) = 4.984, p = 0.028, η^2^ = 0.044), with better performance in those infants with lower MSEL.

At 2 years, outcome groups did not differ in performance (F(3,97) < 1). Performance varied with trial type (F(3, 297) = 46.27, p < 0.001) but there was no interaction type x group (F(9,297) < 1). When adding age, sex and MSEL as covariates, this yielded a main effect of sex (F(1,92) = 5.46, p = 0.022) with boys performing better than girls and no other significant effects were observed.

### 3.3 Additional analyses on search behaviour

Additional measures were derived to further investigate the origin of the superior search performance at 9 and 15 months (see SOM). Briefly, no group differences in biases to orient to a particular side of the screen (left vs right, top vs bottom) were found and biases did not relate to target hit performance. Better performance in the HR-ASD group was not due to the other groups being less accurate in aiming for the target (i.e. landing on the neighbouring AOIs instead). Finally, the amount of time spent on the target, when reached, although longer than the time spent on each distractor visit, did not differ between groups (see SOM for details).

### 3.4 Association with dimensional measures of ASD, ADHD and anxiety symptoms

Social communication symptoms measured using the ADI and SCQ were significantly or marginally correlated with search performance at 9 months and 15 months (Table 3). Since outcome groups differed also in the severity of co-occurring symptoms (ADHD, anxiety; see Table 1), we asked whether performance in the visual search tasks specifically relates to ASD symptoms or more generally to early emerging psychopathology. We found no evidence of significant association between hit performance and traits of ADHD or anxiety based on the results of bivariate correlations reported in Table 3. The patterns of correlations were similar when restricting the results to the HR group only (see Table S1). Number of valid trials at 9 months, however, was significantly associated with ASD, ADHD and anxiety symptoms (rs > 0.18, ps < 0.05; Table 3). We subsequently ran partial correlations controlling for co-occurring ASD symptoms using the parent-rated SCQ, and the associations between the number of valid trials and ADHD or anxiety symptoms were no longer significant (rs < 0.08, ps > 0.37). We also tested the association between parent-rated SCQ and visual search performance at 9 months, controlling for the effects of ADHD and anxiety symptoms separately. The correlation remained significant when controlling for ADHD symptoms (r = 0.17, p = 0.05), but not when controlling for anxiety symptoms (r = 0.14, p = 0.12).

**Table 3:** Bivariate correlations between task performance (number of valid trials and first-look hits) measured at 9 and 15 months and continuous measures of ASD, ADHD and anxiety symptoms and Mullen scores measured at 3 years of age, in the whole sample (see SOM for the same analysis restricted to the HR group).

	**ADI** **Social**	**ADI** **Comm**	**ADI** **RRB**	**ADOS** **Social**	**ADOS** **RRB**	**SCQ**	**CBC** **ADHD**	**CBC** **Anxiety**	**MSEL** **ELC**
**Valid 9m** p N	**.225** **.009** **135**	**.214** **.013** **135**	**.214** **.013** **135**	.112 .194 135	**.237** **.006** 135	*.155* *.075* *132*	**.175** **.050** **127**	.092 .302 127	−.031 .725 135
**Hits 9m** p N	**.229** **.007** **135**	*.162* *.060* *135*	.108 .212 135	.047 .589 135	.132 .128 135	**.211** **.015** **132**	.049 .584 127	.061 .495 127	−.009 .320 135
**Hits 15m** p N	**.230** **.009** **127**	**.201** **.023** **127**	*.150* *.092* *127*	−.007 .944 127	.075 .400 127	*.166* *.064* *125*	.026 .779 121	.022 .810 121	−.023 .794 127

Valid – number of valid trials; Hits – proportion of first-look towards targets; ADI – Autism Diagnostic Interview with subscales of social, communication (Comm) and Restricted and Repetitive Behaviour subscales (RRB); ADOS – Autism Diagnostic Observation Schedule; SCQ – Social Communication Questionnaire; MSEL – Mullen Scales for Early Learning Early Learning Composite; CBC – Child Behavior Checklist; Significant associations (p < .05) are indicated in bold and marginal associations (p < .1) in italics.

## 4 Discussion

The first key question we addressed in this paper was whether superior visual search performance during infancy is observed in HR siblings who go on to receive a later ASD diagnosis (HR-ASD). At 9 and 15 months but not at 2 years of age, visual search performance differentiated those infants who met clinical criteria for ASD at 3 years of age from the high-risk infants without a diagnosis and from low risk controls, with superior search performance observed in the HR-ASD group (Fig. 1). These findings extend our previous report of an association between 9-month old search performance and dimensional measures of ASD symptoms at 2 years of age (Gliga et al., 2015a) and establish superior visual search as an antecedent of autism spectrum disorders, i.e. a marker associated with later diagnosis, but which manifests before the onset of clinical diagnostic symptoms (Johnson et al., 2015).

A second key aim was to better characterise the mechanisms underlying the HR-ASD infants’ superiority in the visual search task. Because superiority is demonstrated in the first-look performance, differences in oculomotor control could not explain the findings. This conclusion was backed-up by our follow-up analysis of the direction of the first look, which showed that poorer performance in the other outcome groups was not due to them just missing the target due to poor oculomotor control. Previous research had suggested that less strong side biases in ASD may help their visual search (Keehn and Joseph et al., 2016) but we found that this cannot explain performance in our task. It has also been suggested that superior search results from better discrimination of target and distractor elements, given ASD participants perform better especially when target and distractors were very similar to each other (Kaldy et al., 2011, O'Riordan et al., 2001). Target type did affect performance (with higher rates of target hits for “O” and “S” targets than for “+” and “V” targets) but target type did not moderate group differences in performance in our study. This does not in itself refute the hypothesis of superior discrimination ability. More fine-grained variation of target/distractor differences or direct assessments of discrimination ability will better address this hypothesis, in the future.

Target detection has also been suggested to vary with arousal levels (Aston-Jones and Cohen, 2005). Blaser et al. (2014) found that during a visual search task, toddlers with autism deployed greater pupil dilation in response to the stimuli, an index of increased arousal; these authors also described an association between larger pupil diameter and superior target hit performance (Blaser et al., 2014). Since the relationship between arousal/pupil dilation and performance is U-shaped (McGinley et al., 2015), with too little or too much arousal associated with poor task performance, the above findings suggest that ASD participants, and not the controls, were in an optimal state of arousal for visual search. Stimulus presentation in our task was too short to measure pupil dilation (1.5 s compared to the 4 s used by Blaser et al.), but we did observe that HR-ASD infants were more attentive to the task than the other groups, spending more time looking at the visual search stimuli. However, this measure of attention did not relate to search performance per se, suggesting that two partially independent processes may account for the atypical attention and perceptual abilities associated with ASD. Interestingly, some have suggested that arousal merely amplifies pre-existing individual differences in information processing (Eldar et al., 2013). Thus, it remains an open question whether perception or arousal-based models better explain the ASD advantage in visual search (and the emergence of ASD symptoms).

The developmental change in the HR-ASD advantage during the first 2 years of life is intriguing, especially given that others have reported superior search later in development, including in 2-year-olds with ASD (Kaldy et al., 2011), an age at which we observed no group differences. One important difference between the Kaldy et al. task and ours is in the nature of the target/distractor differences. It was in the conjunction task (when both colour and shape highlighted the targets) that the ASD group excelled in their study (Kaldy et al., 2011). Our task is more akin to a singleton search, since the O and S targets were unique in the display of Xs in having curved lines, the + differed in line orientation and the V had no line crossing. As Fig. 1 suggests, all groups *except the HR-ASD group* improved in performance between 15 and 24 months, “catching-up” with the HR-ASD group. It is thus possible that the development of the visual system eventually masks group differences in simpler tasks and that more difficult searches (such as conjunction searches) are needed to reveal ASD superiority later on. The less prominent developmental change in the HR-ASD performance parallels findings of reduced developmental progressions of structural connectivity (Wolff et al., 2012, Wolff et al., 2015) and suggests decreased plasticity in the HR-ASD group.

Finally, although we demonstrate an association between superior visual search at 9 and 15 months and the severity of ASD symptoms at 3 years of age, no association with ADHD or anxiety symptoms was found. Many of the previously identified infant markers of ASD are based on impairments common to multiple neurodevelopmental outcomes (Jones et al., 2014) and it was suggested that common neurodevelopmental disorders may stem from common genetic etiology (Pettersson et al., 2013). Yet, superior perception had been singled out as a unique feature of ASD (Happé and Frith, 2006). To date, visual search paradigms have seldom been used in ADHD research, except to show poorer search performance in children with this condition (Hazell et al., 1999, Karatekin and Asarnow, 1998, Mason et al., 2004, Mason et al., 2003). In another study, participants with ASD, but not participants with ADHD showed detail-focused drawing styles (Booth et al., 2003). However, detail-focused or analytic processing were found to be associated with negative mood, in individuals with depression or anxiety (e.g. Derryberry and Reed, 1994, Gasper and Clore, 2002, Hesse and Spies, 1996) and a recent study in adults reported an association between increased anxiety and improved letter detection (Berggren et al., 2015). In contrast to some of these studies, we did not find an association between search performance and either parent-report ADHD or anxiety symptoms, while performance associated with various parental reports of ASD symptom severity (the ADI, the SCQ). Given that parental reports of behavioural atypicalities tend to be highly correlated across dimensions (e.g. the ADI and the CBCL), the differential association between superior search and ASD symptoms is noteworthy.

Although the specificity of this antecedent marker will increase its value in future clinical work, it also raises a significant challenge. While more domain general early markers are being identified, it remains unclear why they impact on the emergence of particular developmental milestones, such as initiation of social interaction or eye-contact, i.e. those ASD traits measured by the ADI/SCQ. While the factors mediating the relationship between early visual attention and perception and later ASD symptoms are yet to be identified, our findings, especially the dynamic changes in perception and its association to ASD symptoms, suggest that answers to these questions are most likely to emerge from research into early development.

## Conflict of interest

None.
