# Supplementary material for: Visual search performance in infants associates with later ASD diagnosis
Source: Dev Cogn Neurosci. 2016 Sep 30;29:4–10. doi: 10.1016/j.dcn.2016.09.003 (PMC6675871; doi:10.1016/j.dcn.2016.09.003)
Supplement: Supplementary file 1 [file mmc1.docx]

**Supplementary Online Material (SOM)**

Figure S1. Differences in search performance between the four trial types, at all ages in all participants contributing data to that age group.

**9 months 15 months 2 years**


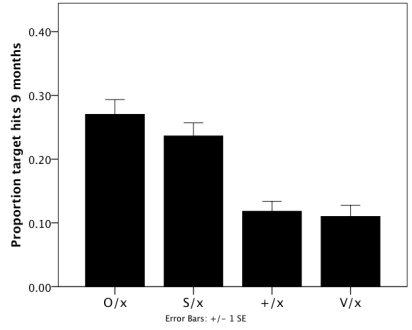

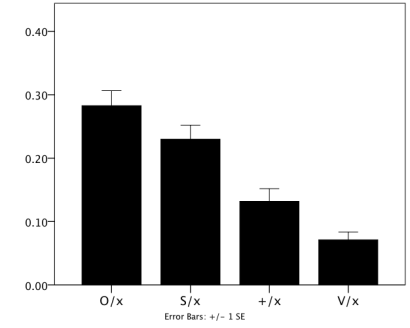

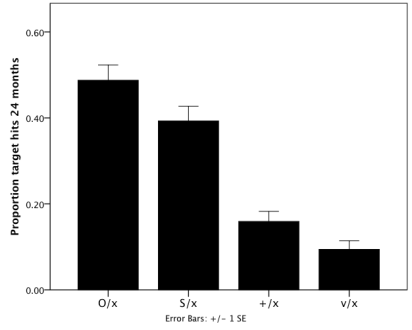


Figure S2. The number of valid trials and the proportion of targets hits do not relate to each other, despite both differentiating between outcome groups.


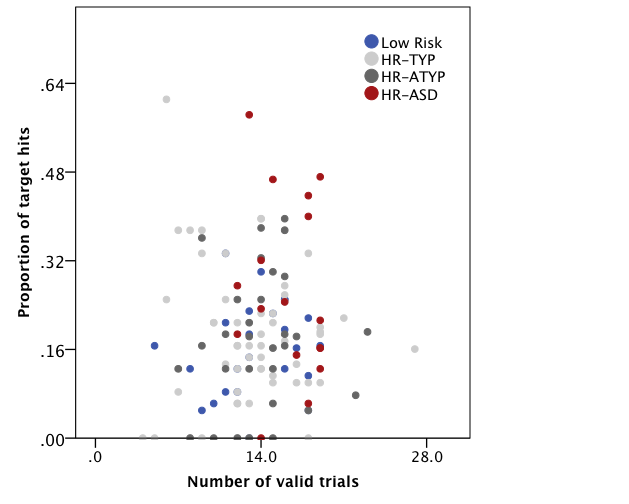


Figure S3. Area of interests (AOI) on visual search stimuli for left (x1, x8), right (x4, o5), top (x6, x7) and bottom (x2, x3) regions.


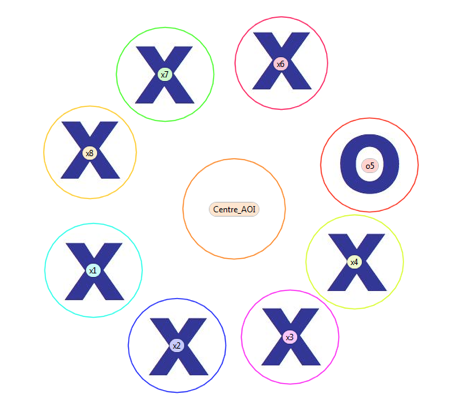


Table S1. Bivariate correlations between task performance (number of valid trials and first-look hits) and continuous measures of ASD, ADHD and anxiety symptoms measured at 3 years of age, in the HR group only.

|  | ADI Social | ADI Com | ADI RRB | ADOSSOC | ADOS RRB | SCQ | CBC ADHD | CBC Anxiety | MSEL ELC |
| --- | --- | --- | --- | --- | --- | --- | --- | --- | --- |
| Valid 9 months  p  N | **.211**  **.027**  **110** | **.181**  **.058**  **110** | **.202**  **.035**  **110** | .144  .132  110 | **.214**  **.025**  110 | .122  .215  106 | .147  .142  102 | **.195**  **.049**  **102** | .005  .961  110 |
| Hits 9 months  p  N | **.217**  **.023**  **110** | .132  .170  110 | .082  .396  110 | .056  .561  110 | .080  .406  110 | .**184**  **.062**  **106** | .027  .784  .102 | .087  .384  102 | .057  .556  110 |
| Hit 15 months  p  N | **.209**  **.033**  **104** | .154  .118  104 | **.**121  .220  104 | .010  .992  104 | .039  .691  104 | .115  .250  101 | -.032  .752  97 | -.007  .944  97 | .015  .883  104 |

Valid – number of valid trials; Hits – proportion of first-look towards targets

**Supplementary methods**

**Participants**. For 111 of 116 children with an older sibling with a community clinical diagnosis of ASD (hereafter probands), parents had completed the Development and Wellbeing Assessment (DAWBA; (Goodman, Ford, Richards, Gatward, & Meltzer, 2000) and/or the Social Communication Questionnaire (SCQ; (Rutter, Bailey, & Lord, 2003). Seventy-seven probands met criteria on both the DAWBA and SCQ. While a small number scored below threshold on the SCQ (n = 8), no exclusions were made due to meeting threshold on the DAWBA and expert opinion. For 19 probands, confirmation of local clinical diagnosis was only available for the SCQ. For 5 probands, neither measure was available aside from parent-confirmed community clinical ASD diagnosis. Screening for possible ASD in the older siblings of the LR infants was undertaken using the SCQ, with no child scoring above the instrument cut-off for ASD (>15) (one missing). Medical history review confirmed a lack of ASD within first-degree relatives.

**Supplementary analysis**

**Analysis accounting for the participation in the intervention programmes**

For all ANOVAs, we began by including two binary terms “treatment” (treated vs not-treated) and “recruited (recruited for intervention vs not recruited for intervention) as covariates. As we were not interested in investigating the effects of the RCT intervention, we only carried out this analysis to rule out any confounding contributions of these variables.

This will be done as such:

1. To take into account any differences due to different sampling of the groups recruited or not recruited for intervention, we will test for a main effect of this factor at all ages.
2. To account for any effect intervention treatment might have had on the visual search performance, we test for a main effect of intervention at 15 months and 2 years (post-intervention); if this effect is significant, the analysis will be re-run without the intervention group.
3. To account for any moderating effect treatment might have had on the relationship between visual search performance and outcome (i.e. if the intervention changed the outcome but did not change visual search performance), we test for a significant treatment x outcome interaction at all ages; if this interaction is significant, we re-run the analysis excluding the intervened group.

Attention to the task. **At 9 months,** outcome groups differed in the number of valid trials (F(3, 129)=2.69, p=0.049), with no significant effect of recruitment into intervention and no significant interactions between outcome and treatment (F(2, 129)<1). **At 15 months**, outcome groups did not differ in the number of valid trials (F(3, 125)<1), and there were no significant main effects of the covariates (F(1, 121)<1), nor any significant outcome x treatment interaction (F(2, 121)<1). **At 2 years**, outcome groups again did not differ in the number of valid trials (F(3, 118)<1), there was no effect of treatment (F(1,118)<1), nor outcome x treatment (F(2, 118)<1) but there was an effect of recruitment into intervention (F(1, 118)=4.01, p =.048), with those children taking part in the intervention having better task attention.

First look hits. **At 9 months,** the main effect of outcome group was at trend (F(3, 119)= 2.503, p=0.063). There were no significant main effects of the covariates (F(1, 119)<1), and outcome x treatment interaction was not significant (F(2, 119)<1). **At 15 months,** main effect of outcome group was not significant (F(3,109)=2.008, p = .117), nor any main effects of the covariates (F(1,109)<1), and no significant outcome x treatment effect was observed (F(2,109)<1). **At 2 years**, no significant main effect of outcome group was observed (F(3, 95)<1), nor were there significant main effects of the covariates (F(1, 95)<1) or outcome x treatment interaction effect (F(2, 95)<1).

**Group differences in attention to the task at 9 months**

Because valid trials were defined as those in which infants fixated the centre of the screen at the beginning of the trial, differences in the number of valid trials might result from how well attention getters succeed in capturing and maintaining infants’ attention to the center of the screen (Table S2). We found no main effect of outcome group for the fixation duration to attention getters (F(3, 135)<1), however groups differed in the number of attention getters that successfully captured infant’s attention (out of a maximum of 32; (F(3, 135)=5.038; p<0.002). Post-hoc results suggest that both the HR-TYP and HR-ASD attended to significantly more attention getters compared to LR (p=0.006, p<0.01. respectively); no other group differences were observed. These differences in orienting to attention getters may reflect differences in general attention on task. We therefore also analysed the amount of looking time to the visual search stimuli themselves. A main effect of group was found (F(3, 135)=3.356; p=0.02), with the HR-ASD group showing longer looking time than the LR group (p<0.01).

Bivariate correlations indicated that the number of valid trials were significantly associated with the total looking time on screen (r=0.468, p<0.01) and the total number of looks towards attention getters (r=0.734, p<0.01). This suggests that all these variables reflect differences in sustained visual attention. Interestingly, measures point to the HR-ASD group being better at attending to the task, at least in terms of the amount of time they spent looking at the screen. To account for any potential impact of these group differences on target hit performance, we included the number of valid trials as additional covariate in all subsequent analyses at this age point.

**Additional analyses at 9 and 15 months**

To further understand the better performance of the HR-ASD group we asked a few further questions. Only valid trials were entered in this analysis, as in the main text. Analysis was cumulated across target types and the number of valid trials was entered as covariate.

Side biases in visual orienting. A weaker left side bias in first look direction was previously reported in the ASD participants (Keehn & Joseph, 2016). We asked whether weaker orienting biases might allow for better performance in the visual search task in our HR-ASD group. We ran separate univariate ANOVAs for 9 and 15 months, comparing the number of first-looks towards the 2 leftmost AOIs as a proportion of left (x1, x8) and rightmost (x4, o5) AOIs, and the number of first-looks towards top AOIs as a proportion of top (x6, x7) and bottom (x2, x3) AOIs (Figure S3). At 9 months, no significant effect of outcome group was found for left vs right AOIs (F(3, 129)=1.105, p=0.350) or for top vs bottom AOIs (F(3, 125)=2.117, p=.101). Similarly, at 15 months, no significant main effect of outcome group was found for either left vs right AOIs (F(3, 113)=1.064, p=.367 or top vs bottom AOIs (F(3, 119)<1) (Table S2). None of the side bias measures correlated with first-look performance at 9 months (ps>.1) or at 15 months (ps>.1).

First look distance to the target. The higher proportion of target hits observed in HR-ASD might result not from them being better at detecting the target, but from better saccadic control (i.e. aiming better towards the target). If that was the case, all other groups might show a higher proportion of first looks directed to locations neighboring the target. We coded locations depending on their distance to the target as 1, 2 ,3 or 4 (with 4 being the location diametrically opposite to the target). A smaller value indicates aiming for AOIs closer to the target. We cumulated these scores across target types, and averaged them for each participant (see Table S2). There was no effect of outcome group, at either 9 months (F(3,133) <1) or 15 months (F(3,124) <1).

Looking time to target and distractors. We wanted to investigate whether the HR-ASD superiority is apparent in both attention-getting and attention-holding mechanisms (Cohen, 1972). Whether only one or both mechanisms are atypical in ASD, will help us estimate their impact these biases have on information sampling. Average visit durations was calculated separately for first looks made to targets or to distractors (Table S2). We ran separate repeated measures ANOVAs at 9 months and at 15 months, with stimulus type (target vs distractors) as within-subject factor. At 9 months, there was a trend main effect of stimulus type (F(1, 113) = 2.85, p=0.094), with longer looking towards the target than the distractor AOIs. There was, however, no significant main effect of outcome group (F(3, 113) < 1), nor an outcome x stimulus-type interaction (F(3, 113) <1). First look hits performance was not associated with visit duration to target hits or distractors at this age (rs<.10, ps>.32). At 15 months, no main effect of outcome group, stimulus type or outcome x stimulus-type interaction were found (Fs (1, 102)<1). Better search performance was associated with shorter visit duration to distractors in the whole group (r=-.248, p<.01) and in the high-risk group only (r=-.324, p<.01), but no associations were observed with visit duration to target hits (ps>.35). Thus, ASD superiority is mainly manifested in attention orienting mechanisms.

Table S2. Descriptive statistics of additional analyses at 9 and 15 months

|  | High risk | | Low risk | |
| --- | --- | --- | --- | --- |
|  | ASD | Atypical | Typical |  |
| **9 months** |  |  |  |  |
| Fixation duration to attention getters (s) | 10.01 (3.99) | 10.19 (6.64) | 9.39 (5.32) | 8.63 (4.14) |
| Number of getters attended/32 | 19.41 (2.85) | 18.94 (3.42) | 17.33 (4.93) | 15.07 (4.58) |
| Fixation duration to stimuli (s) | 18.60 (4.28) | 16.60 (4.95) | 15.44 (5.47) | 13.69 (5.77) |
| Left AOIs bias, M(SD)  N | .63 (.34)  17 | .64 (.39)  30 | .51 (.36)  62 | .50 (.36)  25 |
| Top AOIs bias  N | .57 (.37)  17 | .81 (.32)  29 | .64 (.34)  59 | .70 (.36)  25 |
| Target visit duration (s)  N | .57 (.30)  17 | .63 (.27)  25 | .57 (.24)  54 | .56 (.29)  23 |
| Distractor visit  N | .58 (.17)  17 | .59 (.19)  31 | .55 (.13)  63 | .56 (.16)  27 |
| First look distance to target | 2.32^a^ (.35)  17 | 2.31 (.33)  30 | 2.28 (.47)  62 | 2.35 (.42)  27 |
| **15 months** |  |  |  |  |
| Left AOIs bias  N | .60 (.40)  13 | .61 (.39)  26 | .61 (.35)  57 | .46 (.38)  23 |
| Right AOI bias  N | .60 (.43)  13 | .65 (.39)  28 | .59 (.39)  60 | .66 (.36)  24 |
| Target visit duration (s)  N | .58 (.16)  11 | .51 (.29)  24 | .53 (.25)  54 | .52 (.22)  18 |
| Distractor visit  N | .44 (.15)  13 | .58 (.26)  29 | .52 (.16)  61 | .51 (.16)  25 |
| First look distance to target | 2.34 (.29)  11 | 2.27 (.55)  29 | 2.31 (.46)  59 | 2.25 (.39)  25 |

**References**

Cohen, L. B. (1972). Attention-getting and attention-holding processes of infant visual preferences. *Child Dev, 43*(3), 869-879.

Goodman, R., Ford, T., Richards, H., Gatward, R., & Meltzer, H. (2000). The Development and Well-Being Assessment: description and initial validation of an integrated assessment of child and adolescent psychopathology. *J Child Psychol Psychiatry, 41*(5), 645-655.

Keehn, B., & Joseph, R. M. (2016). Exploring What's Missing: What Do Target Absent Trials Reveal About Autism Search Superiority? *J Autism Dev Disord, 46*(5), 1686-1698. doi: 10.1007/s10803-016-2700-1

Rutter, M., Bailey, A., & Lord, C. (2003). *The Social Communication Questionnaire*. Los Angeles, CA: Western Psychological Services, 2003.
